# Circ-RNF111 aggravates the malignancy of gastric cancer through miR-876-3p-dependent regulation of KLF12

**DOI:** 10.1186/s12957-021-02373-5

**Published:** 2021-08-30

**Authors:** Guoxian Wu, Aimin Zhang, Yinglin Yang, Dongping Wu

**Affiliations:** Department of General Surgery, the First People’s Hospital of Xiaoshan, No. 199 Shixin South Road, Chengxiang Street, Xiaoshan District, Hangzhou, Zhejiang China

**Keywords:** Gastric cancer, Circ-RNF111, miR-876-3p, KLF12

## Abstract

**Background:**

The aberrant expression of circular RNAs (circRNAs) plays vital roles in the advancement of human cancers, including gastric cancer (GC). In this study, the functions of circRNA ring finger protein 111 (circ-RNF111) in GC were investigated.

**Methods:**

Quantitative real-time polymerase chain reaction (qRT-PCR) assay was performed for the levels of circ-RNF111, microRNA-876-3p (miR-876-3p) and krueppel-like factor 12 (KLF12) mRNA. RNase R assay was conducted for the feature of circ-RNF111. Cell Counting Kit-8 (CCK-8) assay, colony formation assay, wound-healing assay, and transwell assay were applied for cell viability, colony formation, migration, and invasion, respectively. Flow cytometry analysis was used to analyze cell apoptosis and cell cycle process. The glycolysis level was examined using specific commercial kits. Western blot assay was carried out to measure the protein levels of hexokinase 2 (HK-2) and KLF12. Dual-luciferase reporter assay and RNA immunoprecipitation (RIP) assay were employed to verify the combination between miR-876-3p and circ-RNF111 or KLF12. Murine xenograft model was constructed for the role of circ-RNF111 in vivo. Immunohistochemistry (IHC) was used for KLF12 level.

**Results:**

Circ-RNF111 was higher expressed in GC tissues and cells than normal tissues and cells. Silencing of circ-RNF111 restrained cell viability, colony formation, migration, invasion, cell cycle process and glycolysis and induced apoptosis in GC cells in vitro. Circ-RNF111 positively regulated KLF12 expression via absorbing miR-876-3p. MiR-876-3p downregulation reversed the impacts of circ-RNF111 silencing on GC cell malignant phenotypes. MiR-876-3p overexpression repressed GC cell growth, metastasis and glycolysis, inhibited apoptosis and arrested cell cycle, while KLF12 elevation weakened the effects. Besides, circ-RNF111 knockdown inhibited tumor growth in vivo.

**Conclusion:**

Circ-RNF111 knockdown relieved the development of GC by regulating miR-876-3p/KLF12 axis.

**Supplementary Information:**

The online version contains supplementary material available at 10.1186/s12957-021-02373-5.

## Introduction

Gastric cancer (GC) is the fourth most usual cancer that endangers human health worldwide [1, 2]. Despite various improvements have been made in diagnosis and treatment in the past few decades, the prognosis of GC is still very poor due to the metastasis and recurrence of tumors [3, 4]. Hence, it is imperative to discover new targets for the diagnosis and therapy of GC.

Circular RNAs (circRNAs) are a class of non-coding RNAs (ncRNAs) formed by back-splicing and implicated in human diseases, especially cancers [5–7]. Moreover, emerging evidence has displayed that circRNAs can act as microRNA (miRNA) sponges to influence gene expression, thereby regulating the biological processes in tumor cells [8, 9]. For example, circ_0067934 facilitated cell invasion and growth and repressed apoptosis in thyroid cancer by elevating CXCR1 through sponging miR-1304 [10]. Circ-ADAM9 aggravated the growth and motility of pancreatic cancer via miR-217/PRSS3 axis [11]. The involvement of circRNAs in GC has also been widely explored [12–14]. CircRNA ring finger protein 111 (circ-RNF111, hsa_circ_0001982) was identified as an oncogene in breast cancer [15], colorectal cancer [16] as well as GC [17]. Even so, the functions and regulatory mechanisms of circ-RNF111 remain largely unknown.

MiRNAs are short ncRNAs and served as tumor promoters or suppressors in GC [18]. Yang et al. suggested that miR-876-3p enhanced the apoptosis and curbed the proliferation of pancreatic adenocarcinoma by targeting JAG2 [19]. Tang et al. declared that miR-876-3p directly targeted KIF20A to slow the carcinogenesis of glioma [20]. Moreover, the downregulation of miR-876-3p was related to the worse outcome of GC patients, and enhanced chemoresistance of GC by targeting TMED3 [21]. Nevertheless, the exact functions of miR-876-3p in GC remain unclarified.

Krueppel-like factor 12 (KLF12) is a member of the KLFs’ family and plays essential roles in various human cancers, including GC [22, 23]. In the study, bioinformatics analysis presented that miR-876-3p included the binding sequences of circ-RNF111 and KLF12, indicating the potential relationships of circ-RNF111, miR-876-3p, and KLF12.

Here, the expression pattern of circ-RNF111 in GC was determined. Moreover, the functions and relationships of circ-RNF111, miR-876-3p, and KLF12 in GC development were investigated.

## Materials and methods

### Tissues acquisition

Thirty-one GC patients at the First People’s Hospital of Xiaoshan were recruited in our research. After the study was permitted by the Ethics Committee of the First People’s Hospital of Xiaoshan and written informed consents were offered by the participants, the tumor tissues and adjacent non-tumor tissues were acquired and stored at – 80 °C before use.

### Cell culture

GES-1 cells and AGS cells were obtained from Procell (Wuhan, China). GC cells (SNU-638) were acquired from Shanghai GuanDao Biological Engineering Co., Ltd. (Shanghai, China). The cells were cultivated at 37°C in DMEM (Sigma-Aldrich, St. Louis, MO, USA) added with 10% FBS (Sigma-Aldrich) and 1% penicillin-streptomycin (Sigma-Aldrich) in a humidified incubator.

### Quantitative real-time polymerase chain reaction (qRT-PCR)

After being extracted via RNAiso Plus (Takara, Dalian, China), the RNA was reversely transcribed into cDNAs with PrimeScript™ RT reagent Kit (Takara) or TaqMan miRNA assays (Applied Biosystems, Foster City, CA, USA). Then, qRT-PCR was conducted through the usage of SYBR Premix DimerEraser (Takara) and related primers (Sangon, Shanghai, China). The expression was estimated with the 2^−ΔΔCt^ way with normalization to GAPDH or U6. The primers included: circ-RNF111: (F: 5′-ACAATCCAGCTGTTCCCTCA-3′ and R: 5′-GGCTCTGGATGCAAAAGGAT-3′); miR-876-3p: (F: 5′-CTGTGGTGGTTTACAAAGTAATT-3′ and R: 5′-GTGCAGGGTCCGAGGT-3’); KLF12: (F: 5′-TGGCAAAGCACAAATGGAC-3′ and R: 5′-CTAAATGGTGAAATTGAACAAGG-3′); GAPDH: (F: 5′-GGAGTCCACTGGCGTCTTCA-3′ and R: 5′-GGTTCACACCCATGACGAAC-3′); U6: (F: 5′-ATTGGAACGATACAGAGAAGATT-3′ and R: 5′-GGAACGCTTCACGAATTTG-3′). To analyze the feature of circ-RNF111, total RNA was exposed to RNase R (3 U/μg; Epicentre, Madison, WI, USA) for 15 min at 37 °C and then circ-RNF111 and GAPDH levels were detected.

### Cell transfection

Circ-RNF111 siRNAs (si-circ-RNF111#1 and si-circ-RNF111#2), circ-RNF111 overexpression plasmid (circ-RNF111), miR-876-3p mimics, miR-876-3p inhibitors (anti-miR-876-3p), KLF12 overexpression plasmid (KLF12), circ-RNF111 shRNA (sh-circ-RNF111), and related controls (si-NC, Vector, miR-NC, anti-miR-NC, pcDNA, and sh-NC) were designed by GenePharma (Shanghai, China). GC cells were introduced with the compositions utilizing Lipofectamine 2000 (Invitrogen, Carlsbad, CA, USA).

### Cell counting kit-8 (CCK-8) assay

Firstly, 5 × 10^3^ cells were seeded into each well of 96-well plates. After 48 h of incubation, 10 μL CCK-8 (Sigma-Aldrich) was supplemented into the well and kept for an additional 2 h. At last, the absorption at 450 nm was measured.

### Colony formation assay

Following indicated transfection, GC cells were seeded into 6-well plates for 14 days. When the colonies were visible, the culture was terminated. Next, the colonies were dyed with crystal violet (Sangon) and quantified utilizing a microscope (Olympus, Tokyo, Japan).

### Flow cytometry analysis

The apoptosis and cell cycle process were examined with Annexin V-FITC/PI Apoptosis Kit (Beyotime, Shanghai, China) based on the manufacturers’ instructions. To examine cell apoptosis, GC cells with various transfections were harvested, washed with PBS (Sangon), and then resuspended in binding buffer. Then, Annexin V-FITC and PI were adopted to dye the cells. For cell cycle process, after the transfected cells were washed, they were fixed with 70% ethanol and then kept with RNase (Solarbio, Beijing, China) in PBS (Sangon) for 1 h. Thereafter, the cells were interacted with PI. The apoptotic rate and cell cycle were assessed with a FACS flow cytometry (BD Biosciences, Franklin Lakes, NJ, USA).

### Wound-healing assay

To determine the migration of GC cells, the transfected GC cells were added into 6-well plates and grown until 100% confluence. Then, the sterile pipette tip was utilized to make a scratch in the well. At 0 h and 24 h, the crosses were recorded.

### Transwell assay

The invasion of GC cells was evaluated with the transwell insert chambers (BD Biosciences) pre-covered Matrigel (BD Biosciences). In short, the transfected AGS and SNU-638 cells in DMEM (Sigma-Aldrich) with serum-free were added into the upper chamber. The bottom chamber was filled with DMEM (Sigma-Aldrich) including 10% FBS (Sigma-Aldrich). Twenty-four hours later, the invaded cells were dyed with crystal violet (Sangon) and quantified utilizing a microscope (Olympus; × 100 magnification).

### Measurement of glycolysis level

The lactate assay kit (Sigma-Aldrich), glucose assay kit (Sigma-Aldrich), and ATP assay kit (Sigma-Aldrich) were employed to test glucose uptake, lactate production, and ATP production levels according to the manufacturers’ protocols.

### Western blot assay

The protein was contained by lysing tissues and cells into RIPA buffer (Beyotime), electrophoresed on SDS-PAGE (Solarbio, Beijing, China) and then blotted onto PVDF membranes (Millipore, Billerica, MA, USA). After blockage in 5% skim milk for 1 h, the proteins were interacted overnight with primary antibodies and secondary antibody (bs-0295M-HRP; Bioss, Beijing, China) for 1.5 h. The bands were visualized with ECL reagent (Beyotime) and band intensity was examined with ImageJ (NIH, Bethesda, MD, USA). The primary antibodies including β-actin (bs-0061R; Bioss), hexokinase2 (HK-2; bs-3993R; Bioss), CyclinD1 (bs-0623R; Bioss), MMP9 (bs-4593R; Bioss), or KLF12 (bs-16783R; Bioss)

### Dual-luciferase reporter assay

The fragments of wild-type (WT) circ-RNF111, or KLF12 3′UTR including miR-876-3p binding sites or mutant (MUT) circ-RNF111 or KLF12 3′UTR lacking miR-876-3p binding sequences were introduced into pmirGLO (Promega, Fitchburg, WI, USA) to generate WT-circ-RNF111, MUT-circ-RNF111, WT-KLF12 3′UTR, and MUT-KLF12 3′UTR, respectively. Next, the generated vectors and miR-NC/miR-876-3p were administrated into GC cells. The luciferase activity was measured utilizing Dual-Luciferase Reporter Assay System (Promega).

### RNA immunoprecipitation (RIP) assay

In brief, after AGS and SNU-638 cells were lysed in RIP buffer, the cell lysates were maintained with protein A/G sepharose beads conjugated with antibody IgG or Ago2. Next, total RNA in immunoprecipitates was subjected to qRT-PCR for the abundance of circ-RNF111, miR-876-3p, and KLF12.

### Murine xenograft model

The BALB/c nude mice from Beijing Vital River Laboratory Animal Technology Co., Ltd. (Beijing, China) were assigned into 2 groups (*n* = 5/group). Sh-NC or sh-circ-RNF111 transfected AGS cells (2 × 10^5^) were suspended in 0.2 mL PBS (Sangon) and then subcutaneously introduced into the flank of the mice. After 7 days, tumor size was examined every 5 days and estimated via the formula: (LengthWidth^2^) × 0.5. At 32 days, the mice were sacrificed and xenograft tumors were weighted. The in vivo study obtained permission from the Ethics Committee of Animal Research of the First People’s Hospital of Xiaoshan.

### Immunohistochemistry (IHC) assay

The expression of KLF12 and ki67 in the xenograft tumor tissues was examined by IHC assay, as previously described [24]. The antibodies against KLF12 (bs-16783R) and ki67 (bs-23103R) were provided by Bioss.

### Statistical analysis

The results from three independent experiments were analyzed using GraphPad Prism 7 and exhibited as mean ± SD. The relationship between miR-876-3p level and circ-RNF111 level or KLF12 level in GC tissues was evaluated by Spearman’s correlation coefficient. Student’s *t* test or one-way analysis of variance was utilized for different analysis. *P* < 0.05 was thought to be significant.

## Results

### Circ-RNF111 was upregulated in GC tissues and cell lines

**Fig. 1:**
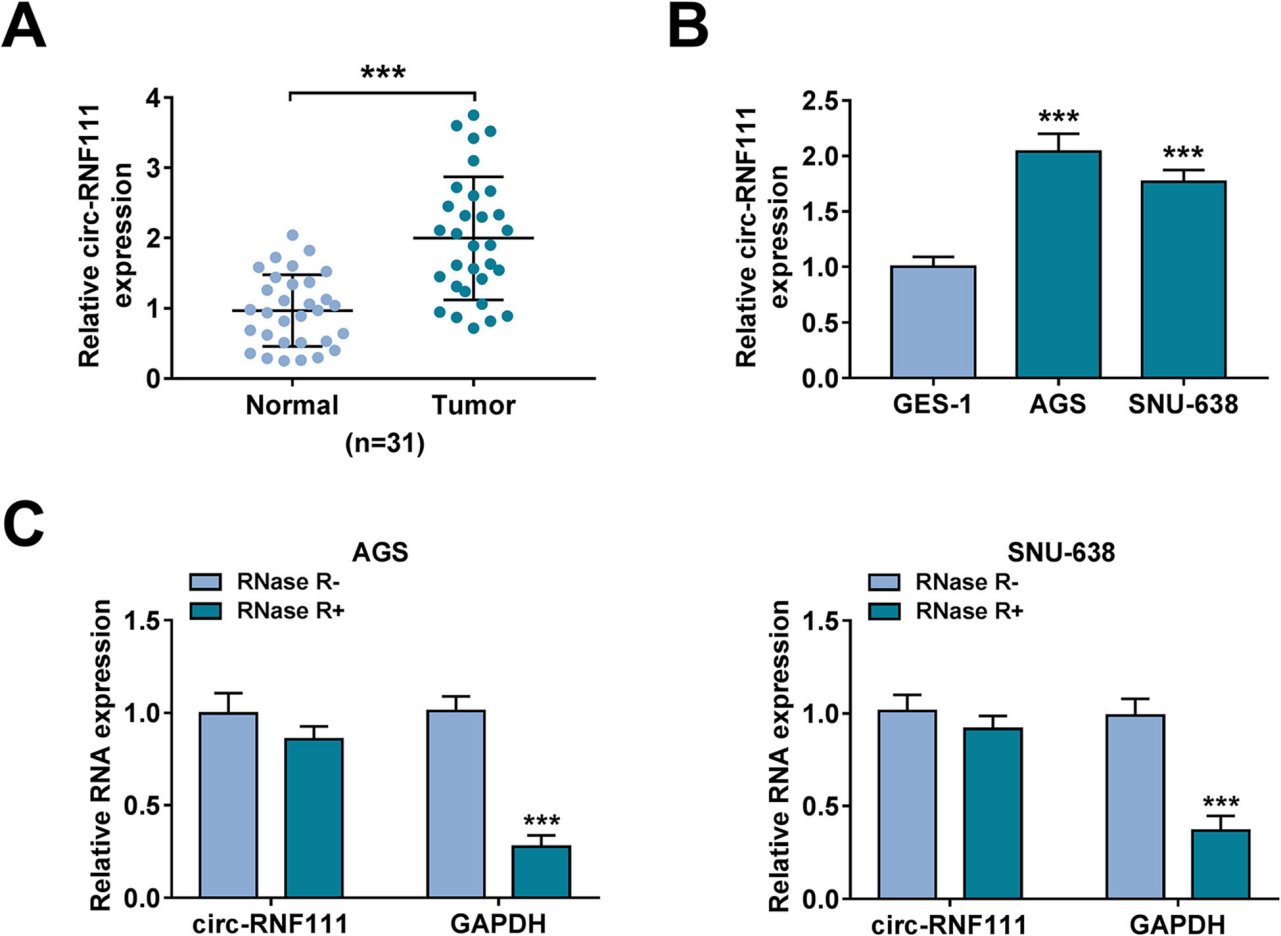
High level of circ-RNF111 in GC tissues and cells. **A** The expression of circ-RNF111 in tumor tissues and normal tissues was determined by qRT-PCR assay. **B** The expression of circ-RNF111 in GES-1, AGS, and SNU-638 cells was detected by qRT-PCR assay. **C** Following total RNA in AGS and SNU-638 cells was treated with or without RNase R, the expression levels of circ-RNF111, and GAPDH were detected by qRT-PCR assay. ***P < 0.001

To explore the effect of circ-RNF111 in GC development, the expression level of circ-RNF111 in GC tissues and normal tissues was determined. The results exhibited that circ-RNF111 was highly expressed in GC tissues compared to adjacent normal tissues (Fig. 1A). Moreover, we found that circ-RNF111 was conspicuously increased in AGS and SNU-638 cells relative to GES-1 cells (Fig. 1B). Besides, RNase R assay showed that GAPDH was digested by RNase R treatment, but circ-RNF111 was resistant to RNase R, indicating that circ-RNF111 was stable (Fig. 1C).

### Silencing of circ-RNF111 suppressed the malignant biological behaviors of GC cells

**Fig. 2:**
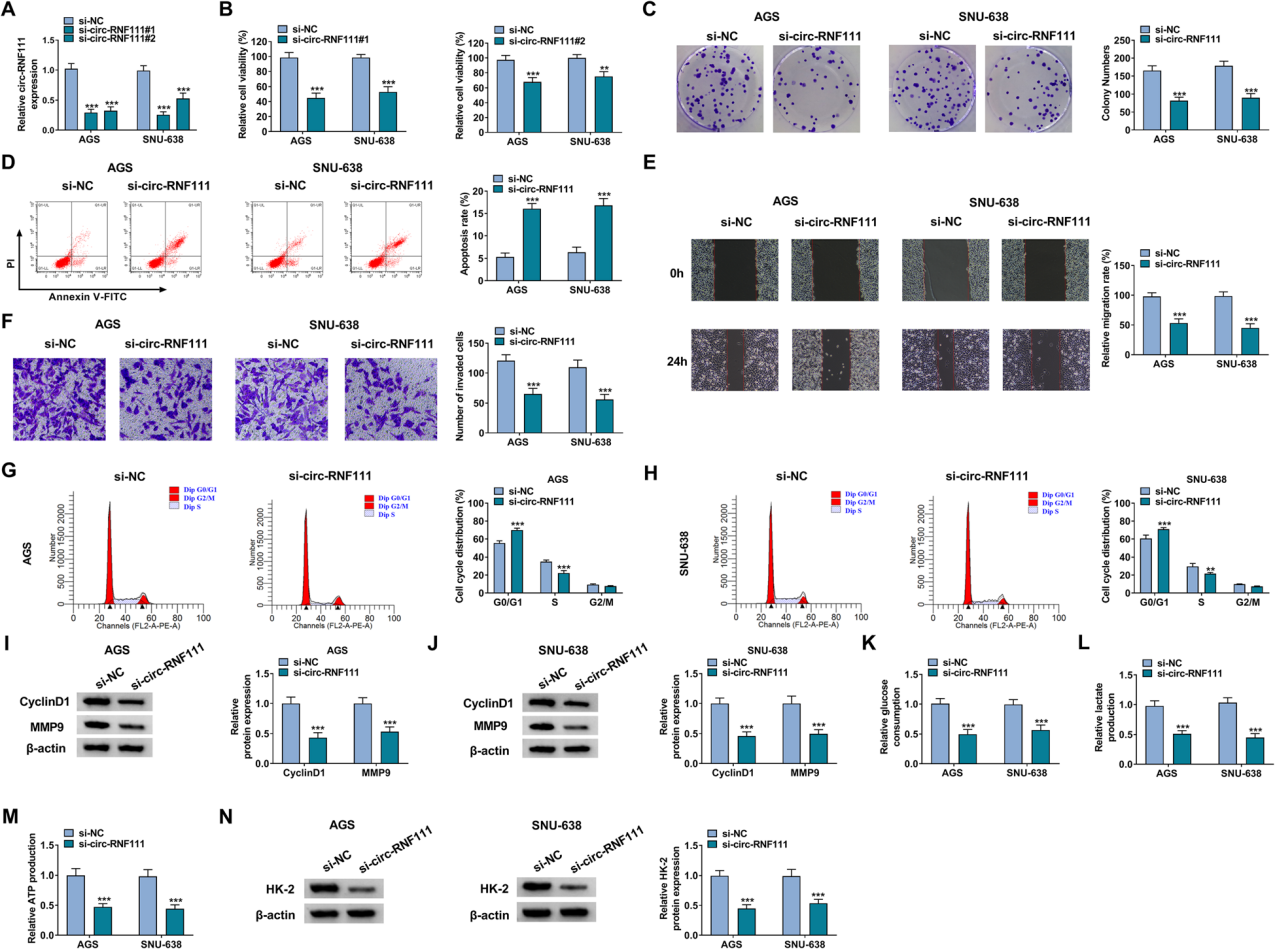
Knockdown of circ-RNF111 repressed the progression of GC cells. **A** The expression of circ-RNF111 in AGS and SNU-638 cells transfected with si-NC, si-circ-RNF111#1, or si-circ-RNF111#2 was detected by qRT-PCR assay. **B** The viability of si-NC, si-circ-RNF111#1, or si-circ-RNF111#2 transfected AGS and SNU-638 cells was assessed by CCK-8 assay. **C**–**L** AGS and SNU-638 cells were transfected with si-NC or si-circ-RNF111. **C** The colony formation, **D** apoptosis, **E** migration, and **F** invasion of AGS and SNU-638 cells were investigated by colony formation assay, flow cytometry analysis, wound-healing assay, and transwell assay, respectively. **G**, **H** Cell cycle process in AGS and SNU-638 cells was analyzed by flow cytometry analysis. **I**, **J** The protein levels of CyclinD1 and MMP9 in AGS and SNU-638 cells were measured by western blot assay. **K**–**M** The levels of glucose uptake, lactate production, and ATP production in AGS and SNU-638 cells were measured with commercial kits. **N** The protein level of HK-2 in AGS and SNU-638 cells was measured by western blot assay. **P < 0.01, ***P < 0.001

To explore the functions of circ-RNF111 in GC, AGS and SNU-638 cells with circ-RNF111 silencing were constructed by transfecting si-circ-RNF111#1 or si-circ-RNF111#2 into AGS and SNU-638 cells. As a result, the transfection of si-circ-RNF111#1 or si-circ-RNF111#2 led to a distinct suppression in circ-RNF111 expression in AGS and SNU-638 cells compared to si-NC control groups (Fig. 2A). The results of CCK-8 assay showed that circ-RNF111 knockdown repressed the viability of AGS and SNU-638 cells relative to control groups (Fig. 2B). Colony formation assay indicated that the colony formation ability of AGS and SNU-638 cells was inhibited by the downregulation of circ-RNF111 in comparison with si-NC control groups (Fig. 2C). Flow cytometry analysis indicated that circ-RNF111 silencing facilitated the apoptosis of AGS and SNU-638 cells in comparison with control groups (Fig. 2D). As illustrated by wound-healing assay and transwell assay, the migration and invasion of AGS and SNU-638 cells were restrained by circ-RNF111 knockdown relative to control groups (Fig. 2E, F). Moreover, circ-RNF111 silencing arrested cell cycle in G0/G1 phase, as analyzed by flow cytometry analysis (Fig. 2G, H). Circ-RNF111 knockdown reduced the levels of cell cycle-related protein CyclinD1 and cell metastasis-related protein MMP9 (Fig. 2I, J). Besides, the impact of circ-RNF111 deficiency on glycolysis was explored. It was found that circ-RNF111 deficiency reduced the levels of glucose consumption, lactate production, ATP production and HK-2 protein in AGS and SNU-638 cells compared to control groups, suggesting the repression of glycolysis in AGS and SNU-638 cells after circ-RNF111 knockdown (Fig. 2K–N). Taken together, circ-RNF111 knockdown suppressed GC cell growth, metastasis and glycolysis, promoted apoptosis, and arrested cell cycle.

### Circ-RNF111 directly targeted miR-876-3p

**Fig. 3:**
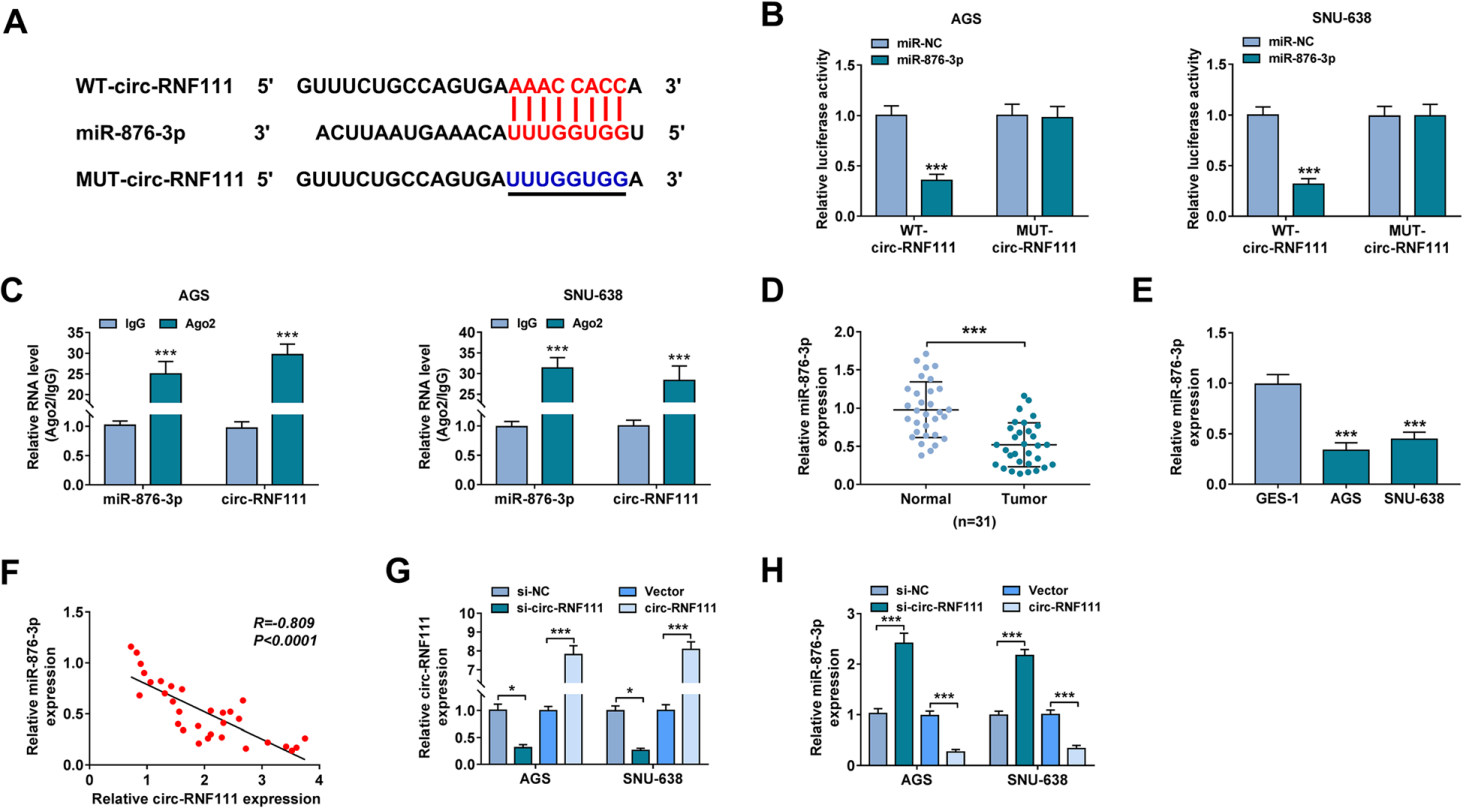
Circ-RNF111 sponged miR-876-3p to regulate miR-876-3p expression. **A** MiR-876-3p contained the binding sites of circ-RNF111. **B**, **C** The interaction between circ-RNF111 and miR-876-3p was verified by dual-luciferase reporter assay and RIP assay. **D**, **E** The expression of miR-876-3p in GC tissues, cells, and normal tissues and cells was determined by qRT-PCR assay. **F** The linear correlation between the levels of circ-RNF111 and miR-876-3p in GC tissues was estimated. **G**, **H** After AGS and SNU-638 cells were administrated with si-NC, si-circ-RNF111, Vector, or circ-RNF111, the levels of circ-RNF111 and miR-876-3p were detected by qRT-PCR assay. ***P < 0.001

Through analyzing circular RNA interactome, miR-876-3p was found to share the binding sites with circ-RNF111 (Fig. 3A). Dual-luciferase reporter assay was then performed to estimate the relationship between circ-RNF111 and miR-876-3p. The results exhibited that the luciferase activity of WT-circ-RNF111 in AGS and SNU-638 cells was restrained by miR-876-3p transfection, while the luciferase activity of MUT-circ-RNF111 was not affected (Fig. 3B). Thereafter, RIP assay further demonstrated the interaction between circ-RNF111 and miR-876-3p for the abundance of circ-RNF111 and miR-876-3p was elevated in Ago2 pellets relative to IgG control groups (Fig. 3C). Indeed, miR-876-3p level was decreased in GC tissues and cells compared to normal tissues and cells (Fig. 3D, E). Moreover, there was an inverse correlation between miR-876-3p level and circ-RNF111 level in GC tissues (Fig. 3F). Besides, si-circ-RNF111 or circ-RNF111 was successfully transfected into AGS and SNU-638 cells to reduce or elevate circ-RNF111 expression, which was demonstrated by qRT-PCR assay (Fig. 3G). Of note, our results exhibited that circ-RNF111 silencing increased miR-876-3p expression in AGS and SNU-638 cells, while circ-RNF111 overexpression showed the opposite results (Fig. 3H). To summarize, circ-RNF111 directly targeted miR-876-3p to regulate miR-876-3p expression.

### Circ-RNF111 knockdown inhibited the malignancy of GC cells by sponging miR-876-3p

**Fig. 4:**
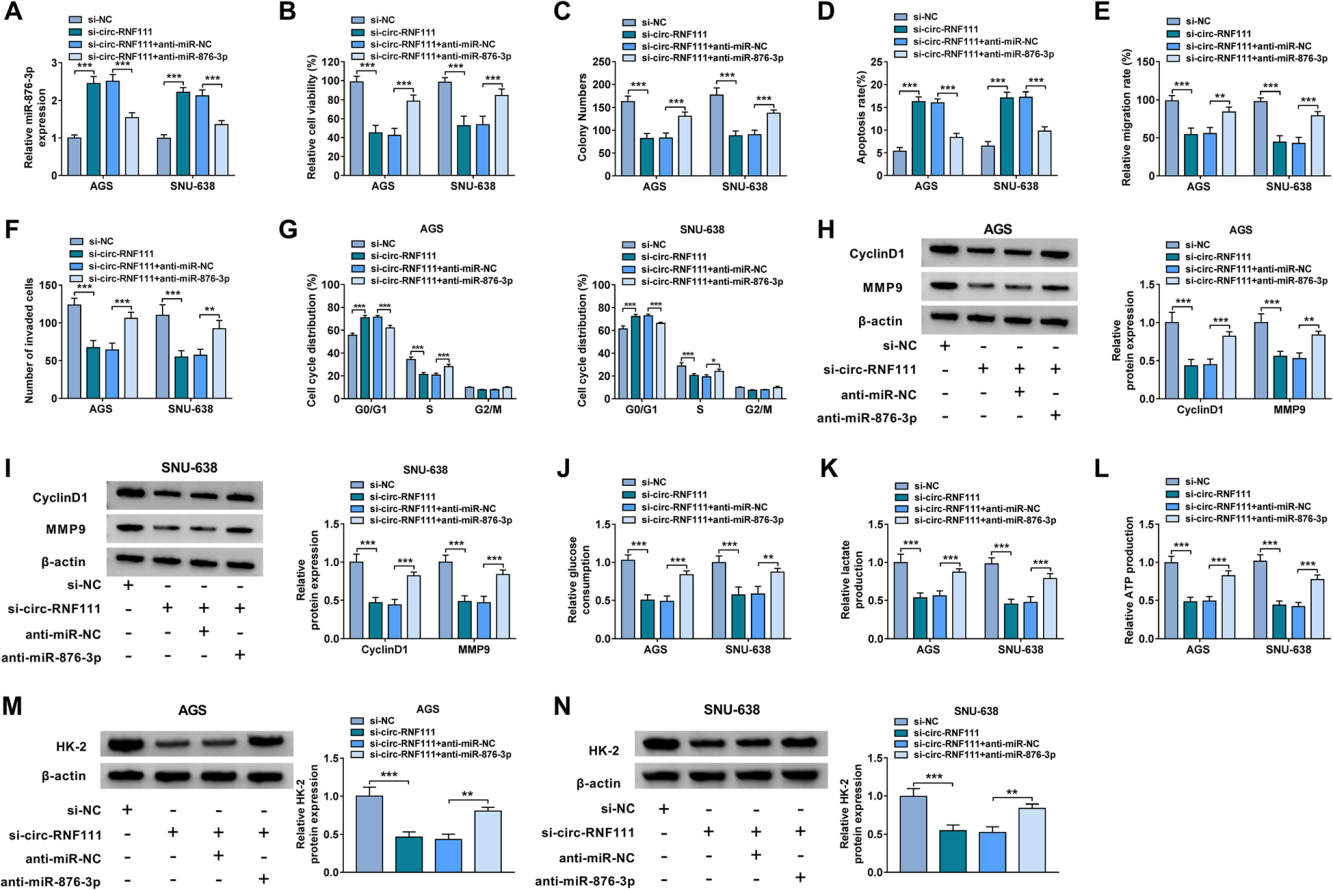
Silencing of circ-RNF111 suppressed the progression of GC cells by targeting miR-876-3p. AGS and SNU-638 cells were transfected with si-NC, si-circ-RNF111, si-circ-RNF111 + anti-miR-NC, or si-circ-RNF111 + anti-miR-876-3p. **A** The expression of miR-876-3p in AGS and SNU-638 cells was detected by qRT-PCR assay. **B** Cell viability, **C** colony formation, **D** apoptosis, **E** migration, and **F** invasion in AGS and SNU-638 cells were evaluated using CCK-8 assay, colony formation assay, flow cytometry analysis, wound-healing assay, and transwell assay, respectively. **G** Cell cycle process of AGS and SNU-638 cells was analyzed by flow cytometry analysis. **H**, **I** The protein levels of CyclinD1 and MMP9 in AGS and SNU-638 cells were measured by western blot assay. **J**–**L** The levels of glucose consumption, lactate production and ATP production in AGS and SNU-638 cells were examined with specific kits. **M**, **N** The protein level of HK-2 in AGS and SNU-638 cells was measured by western blot assay. *P < 0.05, **P < 0.01, ***P < 0.001

Based on the above results, we further investigated whether circ-RNF111 could alter GC cell progression by sponging miR-876-3p through transfecting si-NC, si-circ-RNF111, si-circ-RNF111 + anti-miR-NC, or si-circ-RNF111 + anti-miR-876-3p into AGS and SNU-638 cells. The results showed that anti-miR-876-3p transfection restored si-circ-RNF111-caused upregulation of miR-876-3p level in both AGS and SNU-638 cells compared to anti-miR-NC control groups (Fig. 4A). Furthermore, we found that circ-RNF111 knockdown-mediated inhibitory effects on GC cell viability, colony formation ability, migration, invasion and cell cycle process, and promotional effect on GC cell apoptosis were ameliorated by reducing miR-876-3p (Fig. 4B–G). The effects of circ-RNF111 on CyclinD1 and MMP9 in AGS and SNU-638 cells were also reversed by decreasing miR-876-3p (Fig. 4H, I). Additionally, our results showed that miR-876-3p inhibition reversed the impacts of circ-RNF111 deficiency on glucose consumption, lactate production, ATP production, and HK-2 protein levels in both AGS and SNU-638 cells (Fig. 4J–N). Thus, we concluded that circ-RNF111 silencing repressed GC cell growth, metastasis, and glycolysis and promoted cell apoptosis and cell cycle arrest by targeting miR-876-3p.

### KLF12 was the target gene of miR-876-3p

**Fig. 5:**
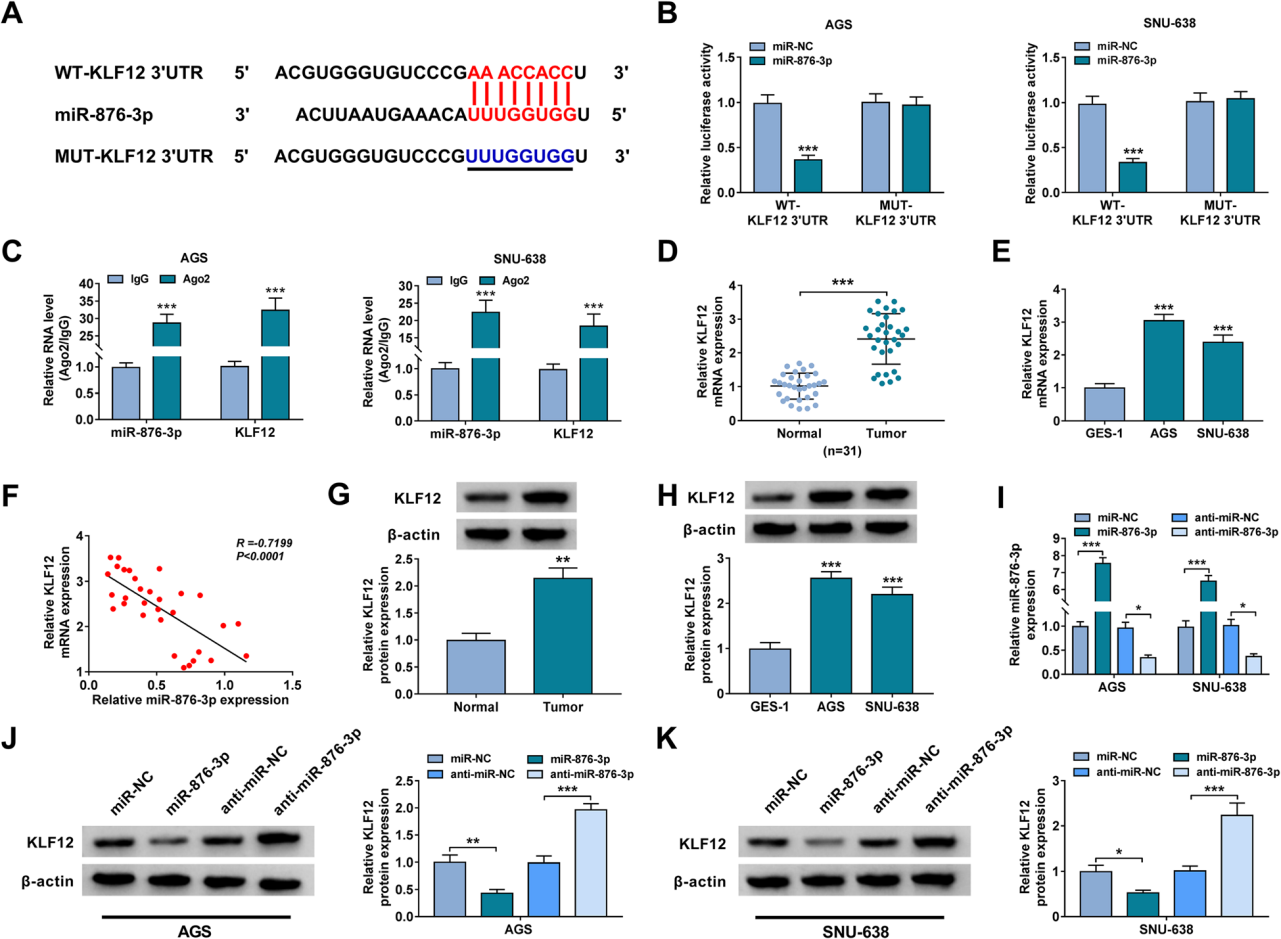
KLF12 acted as the target gene of miR-876-3p. **A** The complementary sequences between KLF12 and miR-876-3p. **B**, **C** The interaction between miR-876-3p and KLF12 was demonstrated by dual-luciferase reporter assay and RIP assay. **D**, **E** The mRNA expression level of KLF12 in GC tissues and cells was detected by qRT-PCR assay. **F** The correlation between the levels of miR-876-3p and KLF12 mRNA in GC tissues was evaluated. **G**, **H** The protein level of KLF12 in GC tissues and cells was tested by western blot assay. **I**–**K** The levels of miR-876-3p and KLF12 protein in AGS and SNU-638 cells transfected with miR-NC, miR-876-3p, anti-miR-NC, or anti-miR-876-3p were detected by qRT-PCR assay and western blot assay, respectively. *P < 0.05, **P < 0.01, ***P < 0.001

To further explore the related regulatory mechanism of circ-RNF111/miR-876-3p in GC development, TargetScanHuman 7.2 was utilized to analyze the potential target gene of miR-876-3p. It was found that KLF12 was the target gene of miR-876-3p (Fig. 5A). Dual-luciferase reporter assay showed that the overexpression of miR-876-3p inhibited luciferase activity of WT-KLF12 3′UTR rather than MUT-KLF12 3′UTR in both AGS and SNU-638 cells (Fig. 5B). RIP assay showed that miR-876-3p and KLF12 were enriched by Ago2 RIP compared to IgG RIP, further verifying the combination between miR-876-3p and KLF12 (Fig. 5C). As expected, KLF12 mRNA level was increased in GC tissues and cells in comparison with normal tissues and cells (Fig. 5D, E). Moreover, KLF12 mRNA level was negatively correlated with miR-876-3p level in GC tissues (Fig. 5F). Western blot assay presented that the protein level of KLF12 was elevated in GC tissues and cells relative to normal tissues and cells (Fig. 5G, H). As shown in Fig. 5I, miR-876-3p transfection increased miR-876-3p expression, while anti-miR-876-3p transfection decreased miR-876-3p expression in AGS and SNU-638 cells. Besides, miR-876-3p overexpression reduced KLF12 protein level and miR-876-3p inhibition elevated KLF12 protein level in both AGS and SNU-638 cells (Fig. 5J, K). Collectively, miR-876-3p directly interacted with KLF12.

### Overexpression of miR-876-3p inhibited GC cell growth, metastasis and glycolysis and facilitated apoptosis and cell cycle arrest by targeting KLF12

**Fig. 6:**
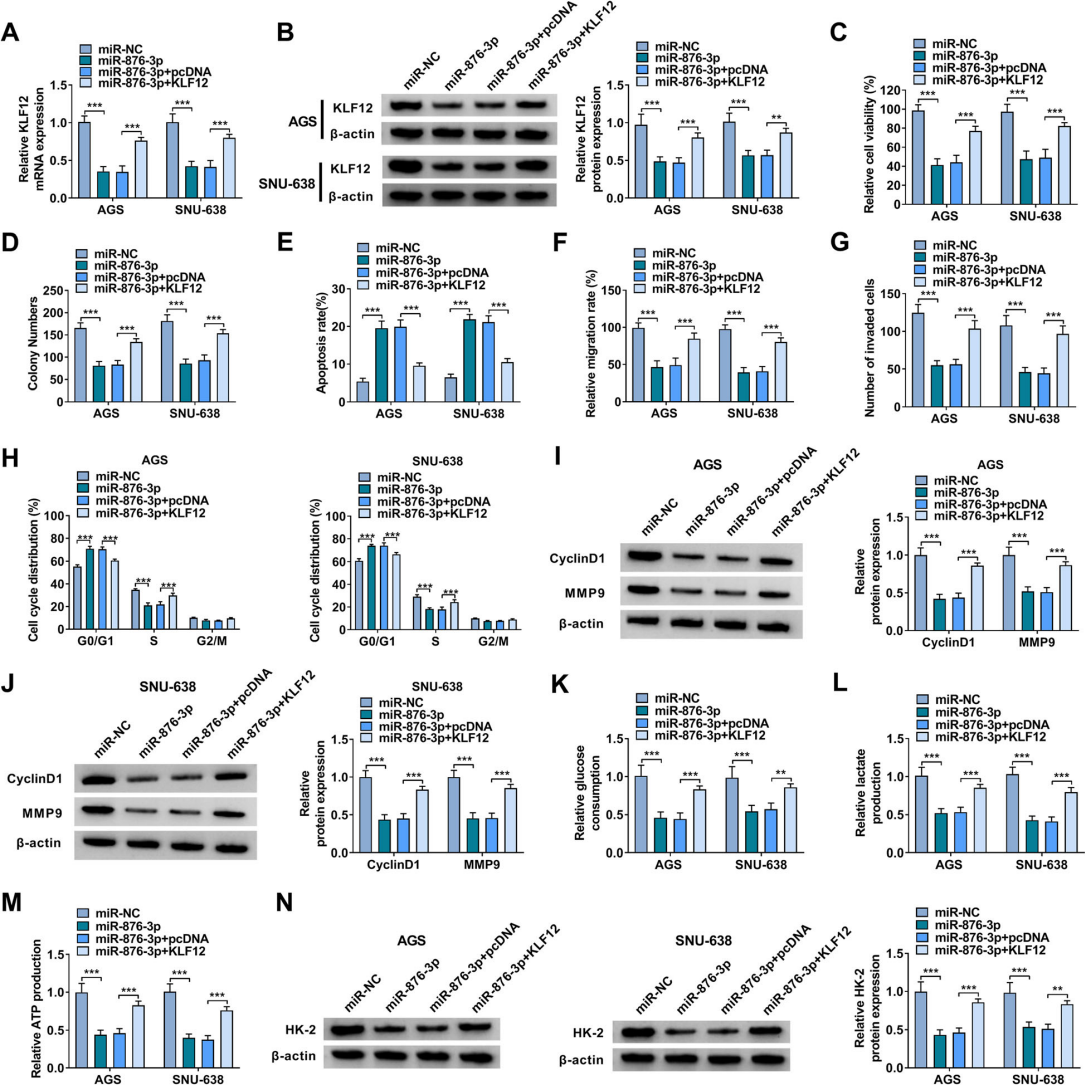
MiR-876-3p overexpression repressed GC cell progression by interacting with KLF12. AGS and SNU-638 cells were administrated with miR-NC, miR-876-3p, miR-876-3p + pcDNA, or miR-876-3p + KLF12. **A**, **B** The mRNA and protein levels of KLF12 in AGS and SNU-638 cells were detected by qRT-PCR assay and western blot assay, respectively. **C** Cell viability, **D** colony formation, **E** apoptosis, **F** migration, **G** invasion, and **H** cell cycle process in AGS and SNU-638 cells were evaluated. **I**, **J** The protein levels of CyclinD1 and MMP9 in AGS and SNU-638 cells were measured via western blot assay. **K**–**M** The levels of glucose consumption, lactate production and ATP production in AGS and SNU-638 cells were examined with kits. **N** The protein level of HK-2 in AGS and SNU-638 cells was measured via western blot assay. **P < 0.01, ***P < 0.001

To investigate the relationship between miR-876-3p and KLF12 in regulating GC progression, rescue experiments were conducted. As shown in Fig. 6A, B, miR-876-3p overexpression reduced the mRNA and protein levels of KLF12 in AGS and SNU-638 cells, while KLF12 overexpression vector transfection rescued the impacts. CCK-8 assay and colony formation assay indicated that miR-876-3p overexpression suppressed the viability and colony formation of AGS and SNU-638 cells, with KLF12 elevation reversed the impacts (Fig. 6C, D). As demonstrated by flow cytometry analysis, miR-876-3p overexpression facilitated AGS and SNU-638 cell apoptosis, while the effect was abrogated by elevating KLF12 (Fig. 6E). The results of wound-healing assay and transwell assay, miR-876-3p upregulation suppressed the capacities of AGS and SNU-638 cells to migrate and invade, whereas these effects were ameliorated by KLF12 overexpression (Fig. 6F, G). The cell cycle process of AGS and SNU-638 cells was arrested by miR-876-3p overexpression, with KLF12 elevation abrogated the effect (Fig. 6H). Western blot assay showed that miR-876-3p overexpression reduced CyclinD1 and MMP9 protein levels in AGS and SNU-638 cells, with KLF12 elevation abrogated the effects (Fig. 6I, J). Besides, overexpression of miR-876-3p reduced the levels of glucose uptake, lactate production, ATP production, as well as HK-2 protein in AGS and SNU-638 cells, while KLF2 elevation abated the impacts (Fig. 6K–N). Taken together, miR-876-3p overexpression suppressed the malignancy of GC cells by targeting KLF12.

### Circ-RNF111 knockdown suppressed KLF12 expression by targeting miR-876-3p

**Fig. 7:**
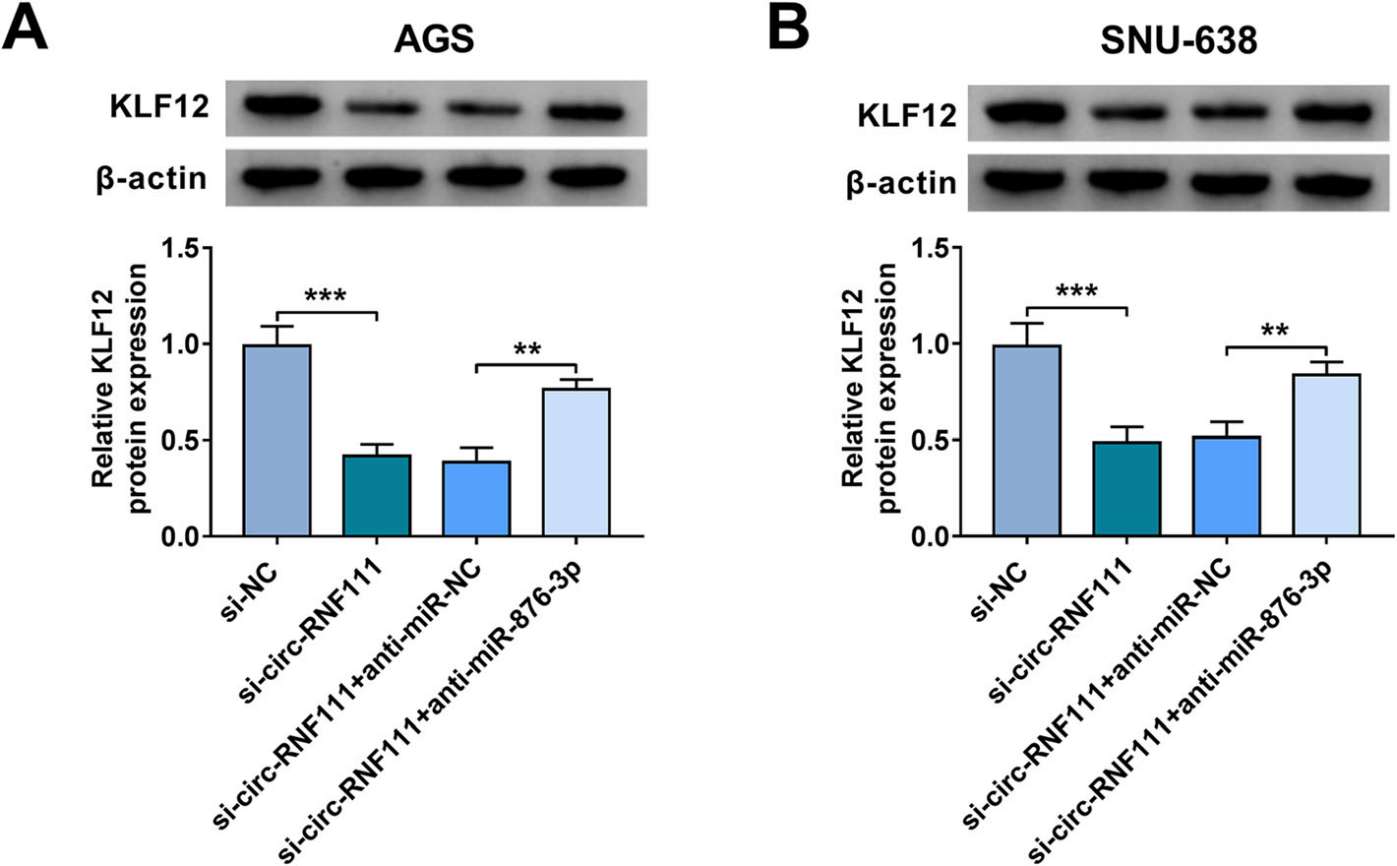
Circ-RNF111 regulated KLF12 expression by sponging miR-876-3p. **A**, **B** After AGS and SNU-638 cells were transfected with si-NC, si-circ-RNF111, si-circ-RNF111 + anti-miR-NC, or si-circ-RNF111 + anti-miR-876-3p, the protein level of KLF12 was measured with western blot assay. **P < 0.01, ***P < 0.001

As exhibited in Fig. 7A, B, circ-RNF111 silencing led to an apparent suppression in KLF12 protein level in both AGS and SNU-638 cells, while miR-876-3p inhibition restored the impact. The findings indicated that circ-RNF111 positively regulated KLF12 expression in GC cells by adsorbing miR-876-3p.

### Knockdown of circ-RNF111 blocked tumor formation in vivo

**Fig. 8:**
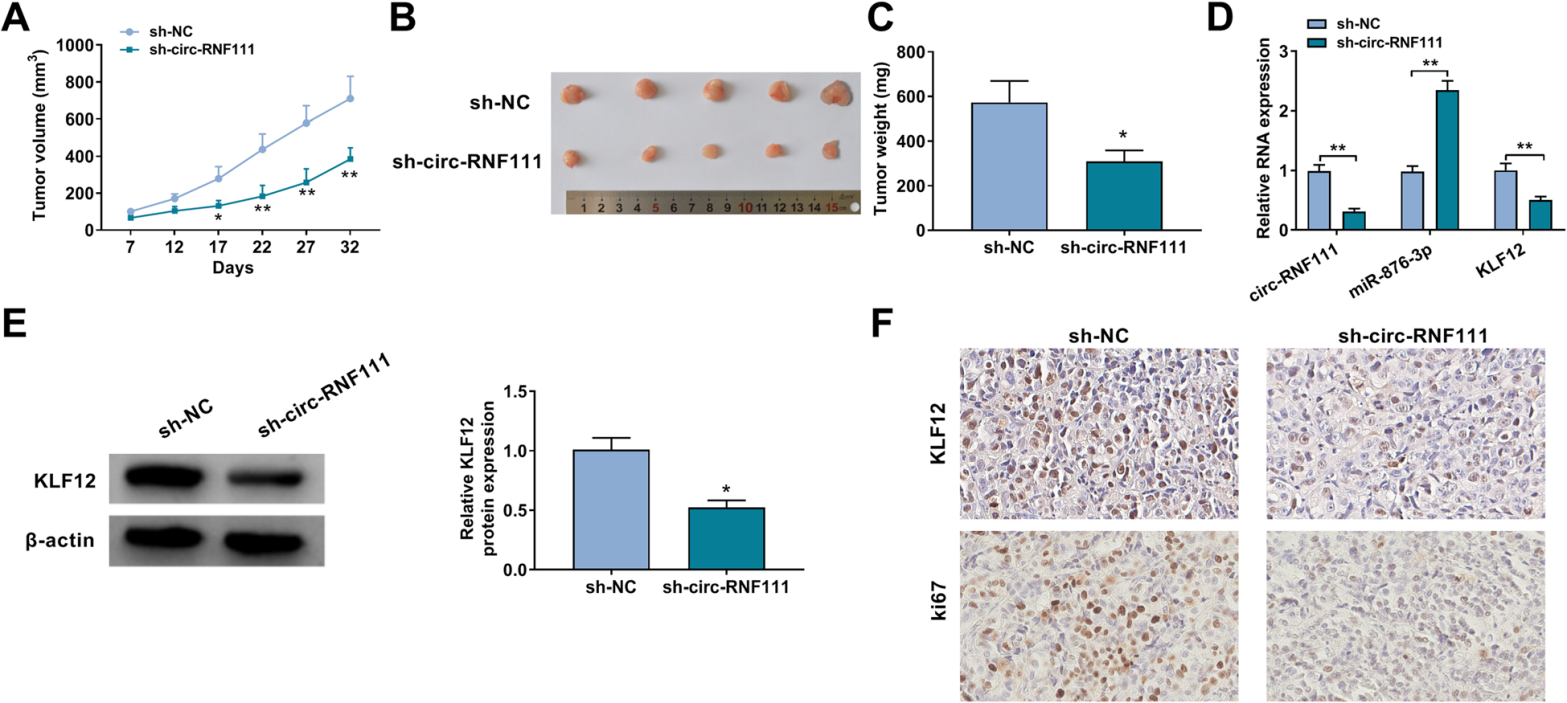
Circ-RNF111 silencing repressed tumor growth in vivo. **A** Tumor volume was monitored. **B** Xenograft tumors were presented. **C** Tumor weight was examined after 32 days. **D** The levels of circ-RNF111, miR-876-3p, and KLF12 mRNA in collected tumors were detected by qRT-PCR assay. **E** The protein level of KLF12 in collected tumors was measured with western blot assay. **F** The levels of KLF12 and ki67 in collected tumors were evaluated by IHC assay. *P < 0.05, **P < 0.01

At last, the functional role of circ-RNF111 in tumor progression in vivo was explored. Our results presented that tumor size and tumor weight were inhibited after circ-RNF111 knockdown compared to sh-NC control groups (Fig. 8A–C). Moreover, the levels of circ-RNF111, KLF12 mRNA, and KLF12 protein were declined and the level of miR-876-3p was enhanced in the tumors in sh-circ-RNF111 groups compared to control groups (Fig. 8D, E). IHC assay also showed that reduced KLF12 and Ki-67 levels in tumors in sh-circ-RNF111 groups compared to sh-NC groups (Fig. 8F). Collectively, circ-RNF111 contributed to tumorigenesis in vivo.

## Discussion

Recently, circRNAs have attracted researchers’ attention for their potential in cancer biology [25]. Substantial evidence implied that circRNAs are engaged in enhancing or repressing GC development. For instance, circ_001653 promoted GC malignancy by upregulating NR6A1 via decoying miR-377 [26]. Circ_0027599 directly targeted miR-101-3p.1, leading to the suppression in GC cell growth and metastasis [14]. Herein, we clarified the functions of circ-RNF111 in GC development. As a result, the upregulation of circ-RNF111 triggered the malignant phenotypes of GC cells. Furthermore, we discovered a novel pathway of circ-RNF111/miR-876-3p/KLF12 in regulating GC development.

Tang et al. manifested that circ_0001982 was overexpressed in breast cancer, and promoted tumor cell growth and invasion through adsorbing miR-143 [15]. Deng et al. demonstrated the oncogenic role of circ_0001982 in colorectal cancer development through sponging miR-144 [16]. Moreover, Wang et al. uncovered that circ-RNF111 sponged miR-27b-3p to aggravate GC cell growth and metastasis and repress apoptosis [17]. Corresponding to the previous studies, we also demonstrated the promotional effect circ-RNF111 in GC. In the present research, circ-RNF111 was elevated in GC. Functionally, circ-RNF111 interference curbed GC cell viability, colony formation, motility, triggered apoptosis and blocked cell cycle process. Besides, tumor cells prefer to obtain energy to meet the need for their rapid growth rather than oxidative phosphorylation and the suppression of glycolysis plays a vital role to hamper tumor progression [27, 28]. Therefore, we explored glycolysis level in GC cells and found that circ-RNF111 silencing decreased glucose uptake, lactate production, ATP synthesis and HK-2 levels, thereby suppressing glycolysis. In addition, to further explore the function of circ-RNF111, the murine xenograft model was established. It was demonstrated that circ-RNF111 silencing restrained tumor formation in vivo. All these findings demonstrated the promotional effect of circ-RNF111 on GC malignancy.

Mechanistically, circ-RNF111 was identified to promote KLF12 expression by serving as the sponge for miR-876-3p in GC cells. MiR-876-3p has been demonstrated to be targeted by circ_0088494 and circTP53 [29, 30], but the association between circ-RNF111 and miR-876-3p was the first to be elucidated. Herein, miR-876-3p was reduced in GC patients MiR-876-3p overexpression repressed GC cell growth, metastasis, and glycolysis and accelerated apoptosis. Moreover, miR-876-3p inhibitor reversed circ-RNF111 knockdown-mediated suppressive roles in GC cell malignant behaviors, indicating that circ-RNF111 influenced GC progression by decoying miR-876-3p. The oncogenic function of KLF12 has been elucidated in several cancers, such as colorectal cancer [31], pancreatic cancer [32], osteosarcoma [33], and nasopharyngeal carcinoma [34]. Moreover, KLF12 could be targeted by miR-618 [35], miR-200a-3p [22], miR-376b-3p [36], and miR-137 [37]. In this work, we discovered that miR-876-3p directly interacted with KLF12 to negatively modulate KLF12 expression in GC cells. The impacts of miR-876-3p overexpression in GC cell malignant phenotypes were abrogated by increasing KLF12, suggesting miR-876-3p could target KLF12 to alter GC progression.

In conclusion, circ-RNF111 was abnormally increased in GC. Moreover, circ-RNF111/miR-876-3p/KLF12 axis could deteriorate the progression of GC by triggering tumor cell growth, metastasis, and glycolysis and curbing apoptosis. Our results might offer key clues to develop an effective treatment method for GC.

## Supplementary Information

**Additional file 1.**

## Data Availability

Not applicable

## References

[1] Jemal A, Bray F, Center MM, Ferlay J, Ward E, Forman D (2011). Global cancer statistics. CA Cancer J Clin..

[2] Siegel RL, Miller KD, Jemal A (2017). Cancer Statistics, 2017. CA Cancer J Clin..

[3] Thrift AP, El-Serag HB (2020). Burden of gastric cancer. Clin Gastroenterol Hepatol..

[4] Ferlay J, Soerjomataram I, Dikshit R, Eser S, Mathers C, Rebelo M, Parkin DM, Forman D, Bray F (2015). Cancer incidence and mortality worldwide: sources, methods and major patterns in GLOBOCAN 2012. Int J Cancer..

[5] Qu S, Zhong Y, Shang R, Zhang X, Song W, Kjems J, Li H (2017). The emerging landscape of circular RNA in life processes. RNA Biol..

[6] Zhao ZJ, Shen J (2017). Circular RNA participates in the carcinogenesis and the malignant behavior of cancer. RNA Biol..

[7] Chen B, Huang S (2018). Circular RNA: an emerging non-coding RNA as a regulator and biomarker in cancer. Cancer Lett..

[8] Zhong Y, Du Y, Yang X, Mo Y, Fan C, Xiong F (2018). Circular RNAs function as ceRNAs to regulate and control human cancer progression. Mol Cancer..

[9] Salzman J, Circular RNA (2016). Expression: Its Potential Regulation and Function. Trends Genet..

[10] Zhang H, Ma XP, Li X, Deng FS (2019). Circular RNA circ_0067934 exhaustion expedites cell apoptosis and represses cell proliferation, migration and invasion in thyroid cancer via sponging miR-1304 and regulating CXCR1 expression. Eur Rev Med Pharmacol Sci..

[11] Xing C, Ye H, Wang W, Sun M, Zhang J, Zhao Z, Jiang G (2019). Circular RNA ADAM9 facilitates the malignant behaviours of pancreatic cancer by sponging miR-217 and upregulating PRSS3 expression. Artif Cells Nanomed Biotechnol..

[12] Liang M, Huang G, Liu Z, Wang Q, Yu Z, Liu Z (2019). Elevated levels of hsa_circ_006100 in gastric cancer promote cell growth and metastasis via miR-195/GPRC5A signalling. Cell Prolif..

[13] Zhang H, Wang X, Huang H, Wang Y, Zhang F, Wang S (2019). Hsa_circ_0067997 promotes the progression of gastric cancer by inhibition of miR-515-5p and activation of X chromosome-linked inhibitor of apoptosis (XIAP). Artif Cells Nanomed Biotechnol..

[14] Wang L, Shen J, Jiang Y (2018). Circ_0027599/PHDLA1 suppresses gastric cancer progression by sponging miR-101-3p.1. Cell Biosci.

[15] Tang YY, Zhao P, Zou TN, Duan JJ, Zhi R, Yang SY, Yang DC, Wang XL (2017). Circular RNA hsa_circ_0001982 Promotes Breast Cancer Cell Carcinogenesis Through Decreasing miR-143. DNA Cell Biol..

[16] Deng Q, Wang CJ, Hao R, Yang QY (2020). Circ_0001982 accelerates the progression of colorectal cancer via sponging microRNA-144. Eur Rev Med Pharmacol Sci..

[17] Wang Z, Jiang Z, Zhou J, Liu Z (2020). circRNA RNF111 regulates the growth, migration and invasion of gastric cancer cells by binding to miR27b3p. Int J Mol Med..

[18] Jiang C, Chen X, Alattar M, Wei J, Liu H (2015). MicroRNAs in tumorigenesis, metastasis, diagnosis and prognosis of gastric cancer. Cancer Gene Ther..

[19] Yang F, Zhao WJ, Jia CL, Li XK, Wang Q, Chen ZL, Jiang Q (2018). MicroRNA-876-3p functions as a tumor suppressor gene and correlates with cell metastasis in pancreatic adenocarcinoma via targeting JAG2. Am J Cancer Res..

[20] Tang J, Xu J, Zhi Z, Wang X, Wang Y, Zhou Y, Chen R (2019). MiR-876-3p targets KIF20A to block JAK2/STAT3 pathway in glioma. Am J Transl Res..

[21] Peng C, Huang K, Liu G, Li Y, Yu C (2019). MiR-876-3p regulates cisplatin resistance and stem cell-like properties of gastric cancer cells by targeting TMED3. J Gastroenterol Hepatol..

[22] Jia C, Zhang Y, Xie Y, Ren Y, Zhang H, Zhou Y (2019). miR-200a-3p plays tumor suppressor roles in gastric cancer cells by targeting KLF12. Artif Cells Nanomed Biotechnol.

[23] Nakamura Y, Migita T, Hosoda F, Okada N, Gotoh M, Arai Y, Fukushima M, Ohki M, Miyata S, Takeuchi K, Imoto I, Katai H, Yamaguchi T, Inazawa J, Hirohashi S, Ishikawa Y, Shibata T (2009). Kruppel-like factor 12 plays a significant role in poorly differentiated gastric cancer progression. Int J Cancer..

[24] Xing S, Qu Y, Li C, Huang A, Tong S, Wu C (2019). Deregulation of lncRNA-AC078883.3 and microRNA-19a is involved in the development of chemoresistance to cisplatin via modulating signaling pathway of PTEN/AKT. J Cell Physiol.

[25] Zhang HD, Jiang LH, Sun DW, Hou JC, Ji ZL (2018). CircRNA: a novel type of biomarker for cancer. Breast Cancer..

[26] Zhou W, Jiang R, Wang Y, Li Y, Sun Z, Zhao H. hsa_circ_001653 up-regulates NR6A1 expression and elicits gastric cancer progression by binding to microRNA-377. Exp Physiol. 2020;105(12):2141–53. 10.1113/EP088399 33006200

[27] Bose S, Le A (2018). Glucose Metabolism in Cancer. Adv Exp Med Biol..

[28] Kroemer G, Pouyssegur J (2008). Tumor cell metabolism: cancer's Achilles' heel. Cancer Cell..

[29] Lou W, Ding B, Wang J, Xu Y (2020). The involvement of the hsa_circ_0088494-miR-876-3p-CTNNB1/CCND1 axis in carcinogenesis and progression of papillary thyroid carcinoma. Front Cell Dev Biol..

[30] Yan S, Wei H, Li Q, Si M, Feng W, Chen Z (2021). CircTP53 promotes colorectal cancer by acting as a miR-876-3p sponge to increase cyclin-dependent kinase-like 3 expression. Cell Signal..

[31] Kim SH, Park YY, Cho SN, Margalit O, Wang D, DuBois RN (2016). Kruppel-like factor 12 promotes colorectal cancer growth through early growth response protein 1. PLoS One..

[32] Hou YS, Li X (2020). Circ_0005273 induces the aggravation of pancreatic cancer by targeting KLF12. Eur Rev Med Pharmacol Sci..

[33] Yuan J, Kang J, Yang M (2020). Long non-coding RNA ELF3-antisense RNA 1 promotes osteosarcoma cell proliferation by upregulating Kruppel-like factor 12 potentially via methylation of the microRNA-205 gene. Oncol Lett..

[34] Song P, Yin SC (2019). Long non-coding RNA 319 facilitates nasopharyngeal carcinoma carcinogenesis through regulation of miR-1207-5p/KLF12 axis. Gene..

[35] Xun J, Wang C, Yao J, Gao B, Zhang L (2019). Long non-coding RNA HOTAIR modulates KLF12 to regulate gastric cancer progression via PI3K/ATK signaling pathway by sponging miR-618. Onco Targets Ther..

[36] Dong MM, Peng SJ, Yuan YN, Luo HP (2019). LncRNA TTN-AS1 contributes to gastric cancer progression by acting as a competing endogenous RNA of miR-376b-3p. Neoplasma..

[37] Du Y, Chen Y, Wang F, Gu L (2016). miR-137 plays tumor suppressor roles in gastric cancer cell lines by targeting KLF12 and MYO1C. Tumour Biol..

