# Supplementary figures and images for: Circ-RNF111 aggravates the malignancy of gastric cancer through miR-876-3p-dependent regulation of KLF12
Source: World J Surg Oncol. 2021 Aug 30;19:259. doi: 10.1186/s12957-021-02373-5 (PMC8404246; doi:10.1186/s12957-021-02373-5)

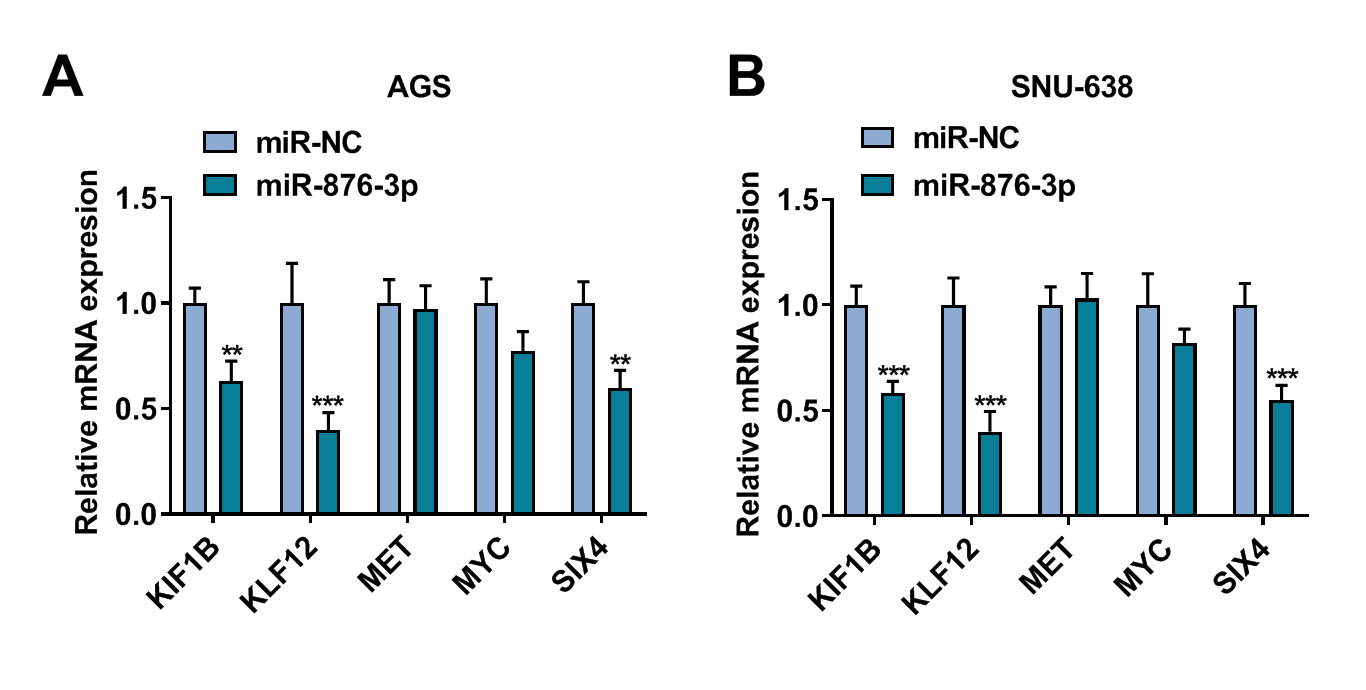

Supplement: Supplementary file 1 — Additional file 1. [file 12957_2021_2373_MOESM1_ESM.tif]
